# Sorcin, a Calcium Binding Protein Involved in the Multidrug Resistance Mechanisms in Cancer Cells

**DOI:** 10.3390/molecules190913976

**Published:** 2014-09-05

**Authors:** Gianni Colotti, Elena Poser, Annarita Fiorillo, Ilaria Genovese, Valerio Chiarini, Andrea Ilari

**Affiliations:** 1 Institute of Biology, Molecular Medicine and Nanobiotechnology, Consiglio Nazionale delle Ricerche, P.le A Moro 5, Rome 00185, Italy; E-Mail: andrea.ilari@uniroma1.it; 2 Institute of Molecular Biology and Pathology, Consiglio Nazionale delle Ricerche, P.le A Moro 5, Rome 00185, Italy; 3 Department Biochemical Sciences “A. Rossi Fanelli”, University Sapienza, P.le A. Moro 5, Rome 00185, Italy; E-Mails: elena.poser@uniroma1.it (E.P.); annarita.fiorillo@uniroma1.it (A.F.); ilaria.genovese@uniroma1.it (I.G.); chiarini.val@gmail.com (V.C.)

**Keywords:** sorcin, calcium, cancer, multi-drug resistance in cancer, endoplasmic reticulum, heart, brain, neurodegenerative diseases, ryanodine receptors, ER stress

## Abstract

Sorcin is a penta-EF hand calcium binding protein, which participates in the regulation of calcium homeostasis in cells. Sorcin regulates calcium channels and exchangers located at the plasma membrane and at the endo/sarcoplasmic reticulum (ER/SR), and allows high levels of calcium in the ER to be maintained, preventing ER stress and possibly, the unfolded protein response. Sorcin is highly expressed in the heart and in the brain, and overexpressed in many cancer cells. Sorcin gene is in the same amplicon as other genes involved in the resistance to chemotherapeutics in cancer cells (multi-drug resistance, MDR) such as *ABCB4* and *ABCB1*; its overexpression results in increased drug resistance to a number of chemotherapeutic agents, and inhibition of sorcin expression by sorcin-targeting RNA interference leads to reversal of drug resistance. Sorcin is increasingly considered a useful marker of MDR and may represent a therapeutic target for reversing tumor multidrug resistance.

## 1. Introduction: Sorcin and the Other Penta-EF Hand Proteins

The EF-hand is a common helix-loop-helix structural motif used by proteins to bind calcium [1]. Most proteins are endowed with an even number of EF-hands, which are usually structurally and functionally paired. Soluble resistance-related calcium binding protein (sorcin) is a calcium binding protein belonging to the penta-EF hand (PEF) family, which includes sorcin, calpains, PDCD6 (formerly called ALG-2), peflin and grancalcin [2].

The members of the PEF protein family participate in different cellular processes, but share several common features: (i) all these proteins have two domains, a flexible and hydrophobic Gly/Pro-rich N-terminal domain, and a C-terminal calcium binding domain containing five EF-hand motifs; (ii) the PEF proteins are dimers, and monomer-monomer association occurs through the unpaired C-terminal EF5 hand; (iii) upon calcium binding, translocation to membranes takes place.

**Figure 1:**
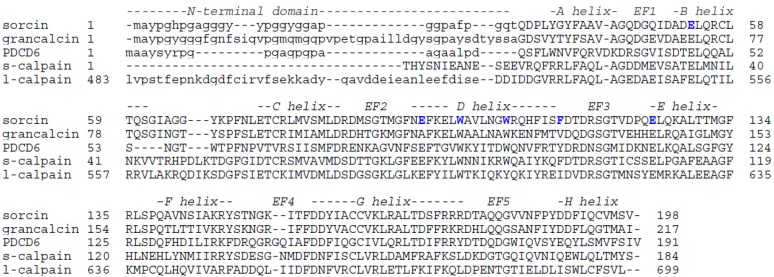
Sequence alignment of human PEF proteins of known crystal structures. Sorcin (NP_003121), grancalcin (NP_036330), PDCD6 (NP_037364), and small and large M-calpain chains are shown. The residues in the N-terminal domain are indicated as lowercase; the residues of the C-terminal calcium binding domain are indicated as uppercase. Structural alignment of EF-hands and of alpha-helices is reported. Glu53, Glu94, Trp99, Trp105, Phe112 and Glu124 are indicated in bold and blue.

The N-terminal domain is often short and flexible, although, in the case of the large subunit of calpains, it can be rather complex, and includes a protease domain. The C-terminal, calcium-binding domain, is rather well conserved among the PEF proteins (Figure 1).

## 2. Sorcin Gene and mRNA Sequence

The gene for sorcin (*SRI*) spans about 21.9 kb of human genomic DNA. The gene is located in chromosome 7q21 and gives rise to at least four alternative transcripts, isoforms A (a primary transcript of 15 kb divided in eight exons and seven introns, transcribed in a 198 aminoacids long protein), B, C and D, yielding shorter forms of the protein, lacking part of the N-terminal domain and/or of the final residues of the C-terminal domain. Most literature results refer to isoform A (22-kDa sorcin), although some works report results on the 19-kDa form of sorcin. A pseudogene (*SRIL*, Sorcin-like) is located in chromosome 4q12.

Sorcin is widely distributed in vertebrates, and its amino acid sequence is highly conserved among species. The protein sequences of the mouse and human sorcin show only eight differences, concentrated in the second half of the protein, and six of them concern phosphorylatable (serine and threonine) residues, an indication that species-specific phosphorylation-dependent regulation of sorcin may take place.

## 3. Sorcin Structure and Mechanism of Activation

**Figure 2:**
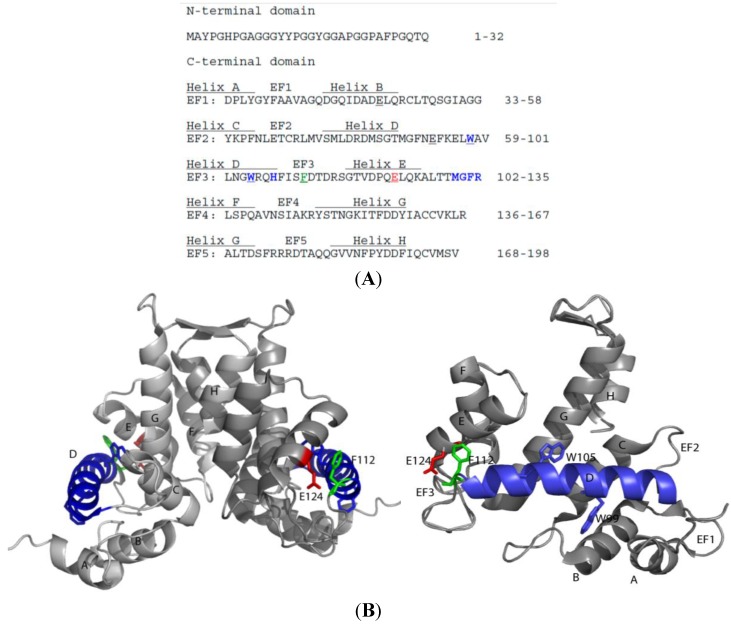
Primary and secondary structure (A) and crystallographic X-ray structure of the human sorcin calcium binding C-terminal domain (B) (PDB: 1JUO). The residues involved in the contact with targets, according to Ilari *et al.*, 2002, are in bold, colored blue. The residues mutated to analyze the mechanism of activation of sorcin (W99, W105, E53, E94, E124, F112) are underlined. The two monomers in the SCBD dimer are depicted in different shades of gray; the D-helix is indicated in blue; W99 and W105 are blue, F112 is green, E124 is red. Right: Only one SCBD monomer is shown, rotated 90° with respect to the dimer shown on the left.

From a structural point of view, sorcin belongs to the small penta-EF-hand family. Sorcin is a homodimer [3,4], although heterodimerization with grancalcin has been described [5]. Each sorcin monomer is formed by two domains, a flexible, glycine-rich N-terminal domain and a C-terminal calcium-binding domain (SCBD). Only the five final residues of the N-terminal domain (residues 28–32) appear to be structured in the apo-form of sorcin [3,6]. The SCBD is globular, and is composed by eight alpha helices (A–H), which form five EF-hands (EF1–5) (Figure 2).

The EF-hands associate into pairs via short β-sheets: EF1 pairs with EF2; EF3 pairs with EF4; the odd EF5 pairs with another EF5, belonging to the contralateral monomer, forming a large part of the dimerization interface. The sorcin dimer is thus the structural protein unit, containing five EF pairs. Helices D and G are long (six turns) and rigid elements that connect different pairs of EF-hands (helix D belongs to both EF2 and EF3; helix G is part of EF4 and EF5), and serve to propagate the information of calcium binding to the whole protein.

SCBD can be divided in two subdomains: EF1-3 (residues 33–134), composed by three EF-hands that bind calcium at micromolar concentration, and EF4-5 (residues 135–198), which does not bind calcium with high affinity, and mediates dimerization. In particular EF4 and EF5 contain many potential phosphorylation sites (see below).

Calcium binding to EF-hands determines the transition from a “closed” structure to an “open” structure [1]. Binding of calcium to EF3, the highest-affinity calcium-binding motif, EF2 and EF1 activates sorcin: Ca^2+^ binding at the EF3 site alters the conformation of loop containing Glu124, and this change is transmitted to EF2 via a movement of the long D helix [4,6]. The canonical structural coupling between EF2 and EF1 allows information transfer to the N-terminus. These organized movements determine exposure to solvent of hydrophobic residues of the d-helix, of the EF loop and of the G helix, with a consequent dramatic decrease of solubility, thus allowing sorcin to translocate from cytosol to membranes, and to bind and regulate a series of target proteins [6,7,8].

## 4. Sorcin Localization, Cell Cycle, and Function in the Cell

Sorcin is expressed in most human tissues, and, according to the MOPED, PaxDb and MaxQB databases, is expressed at high levels in bone, heart, brain, B- and T-lymphocytes, monocytes, kidney, breast and skin. In addition, sorcin is overexpressed in many cancer types (see below). Cell localization of sorcin is dynamic. During interphase sorcin localizes in the nucleus, in the cytosol, in the plasma membranes, at the endoplasmic reticulum (ER) and in ER-derived vesicles localized along the microtubules [9]. These vesicles also contain Ryanodine receptors (RyRs), ER Ca^2+^ ATPase (SERCA), calreticulin and Rab10. In addition, an 18-kDa sorcin variant has been found to be localized at the mitochondrion [10]. During mitosis, sorcin concentrates in the cleavage furrow during late telophase, and at the midbody before cytokinesis [9].

**Figure 3:**
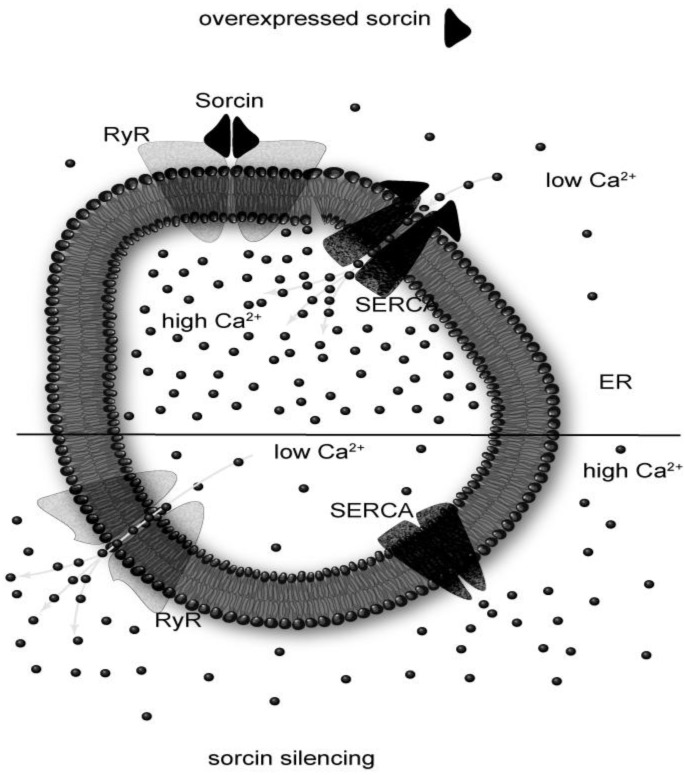
Sorcin inhibits the ryanodine receptor and up-regulates SERCA, increasing calcium load of the endoplasmic reticulum.

In the cytosol, calcium concentration is usually maintained in the range 10–100 nM, because it is actively pumped from the cytosol to the extracellular space, into ER and into mitochondria [11]. In addition, there are many proteins that bind the cation thereby contributing to the calcium buffering process. Sorcin participates in the regulation of calcium homeostasis by different mechanisms. The first mechanism is calcium binding itself: sorcin is able to bind calcium in the micromolar range [7,12]. The second, and most important mechanism is the calcium-dependent binding to calcium channels and to other proteins. Sorcin is able to interact with RyR and sarco(endo)plasmic reticulum Ca^2+^ ATPase (SERCA), located in the ER, and with l-type calcium channel and Na^+^–Ca^2+^ exchangers (NCX), located in the plasma membrane, and to regulate them [13,14,15,16,17]. In particular, sorcin increases calcium accumulation in the ER by activating SERCA and by inhibiting RyR (see also below) (Figure 3), increases dimensions and calcium load of ER-derived vesicles, and is also able to increase mitochondrial calcium concentration [9,18].

High sorcin expression increases ER calcium concentration, can prevent ER stress and the unfolded protein response, and increases escape from apoptosis [9,19,20]. The 18-kDa variant of sorcin is regulated by TRAP1, which controls sorcin folding and expression [19].

Conversely, sorcin silencing activates apoptotic proteases as caspase-3, caspase-12 and GRP78/BiP [20], results in major defects in mitosis and cytokinesis, blocks cell cycle progression in mitosis, increases the number of rounded polynucleated cells and induces apoptosis and cell death [9].

Sorcin interacts with many other targets, in addition to those above mentioned. Many of such targets are serine-threonine kinases, which participate in the regulation of mitosis progression [9]. Sorcin contains several potential phosphorylation sites. Phosphorylation contributes to regulate sorcin activity:sorcin is phosphorylated by polo-like kinase 1 (Plk1), induces Plk1 autophosphorylation, and contributes to Plk1 regulation [9]. cAMP-dependent protein kinase (PKA) and calcium-calmodulin dependent kinase II (CaMKII) phosphorylate sorcin [4,21], altering sorcin binding to RyRs and SERCA and, therefore, calcium homeostasis.

Sorcin has been identified in other vesicles than the ER-dependent ones, such as in nanovesicles containing Annexin A7, released in a calcium-dependent fashion from the erythrocytes [22], and in exosomes from different sources, such as B-cell exosomes [23], mesenchimal stem cell exosomes [24], exosomes from human urine [25,26].

Sorcin has also other possible signaling roles, linking calcium levels with different metabolisms. Sorcin binds to and sequesters the carbohydrate-responsive element binding protein (ChREBP) in the cytosol at low glucose, by interacting with the N-terminal glucose-sensing domain of ChREBP [27]. Following glucose stimulation and calcium influx, sorcin releases ChREBP, which becomes free to translocate to the nucleus.

## 5. Sorcin and Cancer

Sorcin gene is located in chromosome 7, in the same amplicon of other genes involved in the resistance to chemotherapeutic agents in cancer cells (multi-drug resistance, MDR), as the ABC transporters ABCB4 and ABCB1 (Mdr1, or P-glycoprotein 1). The ABC transporters are ATP-dependent efflux pumps with broad substrate specificity, able to pump xenobiotics (such as toxins or drugs) out of cells. Many cancer cells express large amounts of ABCB1, which renders these cancers multi-drug resistant. Sorcin was identified as “resistance-related”, because encoded by a gene co-amplified with P-glycoproteins in multidrug-resistant cells [28], but for years sorcin overproduction was believed to be only the accidental by-product of the coamplification of its gene with P-glycoprotein genes [29].

Sorcin is overexpressed in many human tumors, such as leukemia, lymphoma, adenocarcinoma, gastric, lung, breast, nasopharyngeal and ovarian cancers, and especially in MDR cancers [30,31,32,33,34,35,36,37]. In recent years, an increasing number of data have demonstrated a role of sorcin in MDR, and indicated its role as an oncoprotein. Sorcin is highly expressed in chemoresistant cell lines, significantly up-regulated in a doxorubicin-induced MDR leukemia cell line, K562/A02, over its parent cells, and its overexpression confers MDR. The level of sorcin expression in leukemia patients inversely correlates with patients response to chemotherapies and overall prognosis. Sorcin overexpression by gene transfection (i) resulted in increased drug resistance to a variety of chemotherapeutic agents, including doxorubicin, etoposide, homoharringtonine and vincristine in K562 cells; and (ii) determined drug resistance to vincristine, adriamycin, taxol and 5-fluorouracil in SGC7901 cells, ovarian and breast cancer. On the other hand, inhibition of sorcin expression by sorcin-targeting RNA interference led to reversal of drug resistance in the following cell lines: MDR K562/A02 and sorcin-transfected K562; MCF-7/A02; HeLa; colorectal cancer; and CNE2/DDPls [37,38,39,40,41,42,43]. This demonstrates that sorcin is a useful marker of MDR and may represent a therapeutic target for reversing tumor multidrug resistance.

Sorcin silencing inhibits the epithelial-to-mesenchymal transition in the breast cancer MDA-MB-213 cell line, possibly via E-cadherin and VEGF expression, and reduces breast cancer metastasis, while sorcin overexpression increases migration and invasion *in vitro* [44].

The role(s) of sorcin in the development of the MDR phenotype is currently being studied by several groups. On one side, sorcin expression directly up-regulates ABCB1 expression: sorcin induces P-glycoprotein expression via a cAMP response element (CRE) between -716 and -709 bp of the *ABCB1* gene. In addition, sorcin overexpression induces ABCB1 expression through activation of the CREB pathway, by increasing the phosphorylation of CREB1 and the binding of CREB1 to the CRE sequence of mdr1/p-gp promoter [35].

ER-associated TRAP1, a mitochondrial anti-apoptotic protein upregulated in several human tumors, modulates mitochondrial apoptosis by exerting a quality control on the 18-kDa variant of sorcin, lacking the 30 residues long, glycine-rich N-terminal domain. The transfection of a TRAP1 deletion mutant, localized to the ER, in shTRAP1 cells, increases the expression of mitochondrial sorcin and protects from apoptosis induced by ER stress agents and paclitaxel [19]. Sorcin ability to regulate ER and mitochondrial calcium levels has a possible important impact on the cell fate: sorcin silencing activates caspase-3, caspase-12 and GRP78/BiP, increases mitotic defects, blocks cell cycle progression in G2/M, increases the number of rounded polynucleated cells and induces apoptosis and cell death; on the other end, high sorcin levels increase ER and mitochondrial calcium load [9,20]. In addition, leukemia cell lines overexpressing sorcin showed up-regulation of Bcl-2 and decreased level of Bax [32].

Sorcin thus participates in the prevention of ER stress and of the unfolded protein response, and increases escape from apoptosis [9,19,20], shifting the equilibrium between cell life and cell death towards proliferation in MDR cancer cells overexpressing sorcin.

## 6. Sorcin and the Heart

Cardiac contraction is a complex and fast (800 ms) process, started by the electrical excitation of cardiac myocytes. When the wave of depolarization reaches a cardiomyocyte, voltage-dependent Na^+^ channels open, which results in a rapid cell depolarization. During depolarization, calcium enters the cell via voltage-dependent Ca^2+^channels (VOCC, mainly l-type channels). l-Type Ca^2+^ channels are located primarily in the dyadic space, juxtaposed to sarcoplasmic reticulum (SR) calcium release channels (Ryanodine Receptors: RyRs). Calcium entry via VOCC locally increases Ca^2+^ concentration near RyRs, triggering Ca^2+^release from the SR, a specialized calcium store, similar to ER. This flux further raises the free intracellular [Ca^2+^], allowing Ca^2+^to bind to troponin C and trigger contraction. For relaxation to occur, cytosolic [Ca^2+^] has to decrease, allowing calcium dissociation from the myofilaments. Thus, RyR has to be closed, and calcium has to be transported out of the cytosol, mainly via the SR Ca^2+^-ATPase (SERCA), which takes Ca^2+^back into the SR, and the sarcolemmal Na^+^/Ca^2+^exchanger (NCX) [45].

Sorcin is an important regulator of cardiac contraction, being able to interact with all the main calcium channels-exchangers, responsible for calcium fluxes in cardiac contraction, and to regulate them. Sorcin modulates the l-type VOCC, by interacting with its alpha-1C subunit with its C-terminal domain, slowing Ca^2+^-dependent inactivation and stimulating voltage-dependent inactivation of the calcium currents of the channel [46,47].

In addition, sorcin is able to interact with RyR2, the cardiac RyR, and to strongly inhibit it, in a calcium-dependent fashion [14,48]. In the cardiomyocyte, such RyR inhibition takes place when the local calcium concentration at the surface of SR is increased by calcium-induced calcium release from the calcium store, and results in decreased localized calcium release events (calcium sparks) and reduced global calcium transients (local fast calcium concentration increments). Sorcin reduces calcium flow from RyR2 by decreasing the mean open time and the frequency of open event [6,14,48]. Sorcin also interacts with SERCA, activating it [16]. Sorcin is thus able to increase calcium uptake of SR and to determine negative regulation of its release from SR, in a dose-dependent and calcium-dependent fashion.

Sorcin also activates NCX through a calcium-dependent interaction of the respective C-terminal domains; the overexpression of sorcin in cardiomyocytes has also been associated with increased activity of the Na^+^–Ca^2+^ exchanger [15,17]. Fast association and dissociation rates between sorcin and the calcium binding domains of NCX, enough to allow for a partial cycle of association with (and activation of) NCX and dissociation from (deactivation of) NCX within fractions of seconds and may allow NCX regulation on a “beat to beat” basis [17].

Overall, sorcin regulates the excitation-contraction-relaxation process in the heart, by terminating the calcium-induced calcium release by the SR, and favoring relaxation, by decreasing the cytosolic calcium concentration, with at least three different modalities: it inhibits calcium release from the SR by inhibiting RyR2, it increases calcium entry from cytosol to the SR by activating SERCA2a and favors calcium extrusion from the sarcolemma by increasing NCX activity.

Phosphorylation alters Ca^2+^sensitivity of sorcin, favors its translocation to the sarcoplasmic reticulum (SR) and decreases the capacity to inhibit ryanodine binding to RyR2. Moreover, in the failing heart, sorcin is hyper-phosphorylated and translocation to the SR membrane is increased, possibly resulting in preservation of the SR Ca^2+^content and in improvement of cardiac relaxation [14,16,21].

A mutation in sorcin EF3-hand (F112L) was associated with hypertrophic cardiomyopathy and hypertension. This mutation, as other mutations in the EF3 and/or in the D helix of sorcin, decreases the ability of sorcin to interact with, and to regulate its cardiac targets and to negatively regulate SR calcium release, resulting in complex cardiac alterations [6,49].

In many types of heart failure, cardiac down-regulation of SERCA2a and of RyR2 occurs, resulting in altered cytosolic calcium transients, leading to abnormal contraction. Sorcin overexpression in mice is associated with an increase in cardiac contractility of the normal heart and with a dramatic rescue of the abnormal contractile function of the diabetic heart. These effects could be attributed to an improvement of the calcium transients found in the cardiomyocyte after sorcin overexpression [50,51].

## 7. Sorcin and the Brain

Sorcin expression in the brain is high, about 5-10 times higher than that in the heart. In particular sorcin is one of the most expressed calcium binding proteins in the amygdala, in the prefrontal cortex, in the hypothalamus and in many brain cancers (GeneAtlas U133A, gcrma).

Sorcin is considered a histological marker for malignant glioma [52], and is one of the most expressed proteins in anaplastic astrocytoma, oligodendroglioma and glioblastoma [53,54,55]. Pomeroy and collaborators showed that the level of expression of sorcin represents one of the main markers of poor outcome in embryonal tumors of central nervous system [56].

In addition to the roles of sorcin in the development of brain tumors, deregulation of calcium-mediated signaling has been implicated in many neurodegenerative diseases including Alzheimer's disease (AD) and Parkinson disease (PD). Perturbed ER calcium homeostasis, ER stress, and the consequent accumulation of unfolded protein, are involved in the accumulation and deposits of misfolded proteins in many neurodegenerative diseases, as AD and PD. Several studies have reported alterations of the expression and the function of RyR in human AD-affected brains, in cells expressing familial AD-linked mutations on the β amyloid precursor protein (βAPP) and presenilins (the catalytic core in γ-secretase complexes cleaving the βAPP, thereby generating amyloid β peptides), and in brain of transgenic AD mice models. Alterations in RyR expression and function are associated to AD pathogenesis via the control of: (i) βAPP processing and Aβ peptide production; (ii) neuronal death; (iii) synaptic function; and (iv) memory and learning abilities (for a recent review on this subject, see Del Prete *et al.*, [57]).

Apart from its direct ability to interact with RyR and to inhibit it, thus maintaining calcium load in ER and possibly decreasing the unfolded protein response in the brain (see above), sorcin directly interacts in a calcium-dependent fashion with alpha-synuclein (AS) and presenilin 2 (PS2), two proteins involved in the pathogenesis of PD and AD, respectively, *in vitro*, in cultured cells and in human brain [58,59]. Sorcin binds to the C-terminal region of PS2, a protein that has been suggested to be able to form low-conductance divalent-cation-permeable ion channels in planar lipid bilayers [60], to interact with RyR upon calcium binding, and to regulate calcium homeostasis in the cells [61].

Sorcin is overexpressed in a PD cell model induced by 1-methyl-4-phenylpyridinium ion (MPP^+^) in SH-SY5Y cells [62], and is one of the most differentially expressed proteins in PD *vs.* normal human substantia nigra [63]. Sorcin interacts with the N-methyl-D-aspartate receptor 1 in caudate-putamen nucleus [64]. Further, it interacts with annexins A7 and A11, which affect functions of primary astrocytes [65].

## 8. Conclusions

In the last few years, sorcin has emerged as one of the most intriguing regulators of calcium homeostasis. Although the levels of expression of sorcin is much lower than that of calmodulin, sorcin is differentially expressed in cancers and other pathological conditions and is able to interact with a series of crucial targets and to regulate them. Sorcin regulates calcium channels and exchangers located at the plasma membrane and at the endo/sarcoplasmic reticulum (ER/SR), such as RyR, SERCA, NCX and l-type VOCC, and allows high levels of calcium in the ER (and possibly in the mitochondrion) to be maintained, preventing ER stress and apoptosis.

Sorcin is highly expressed in the heart and in the brain, and is overexpressed in many cancer cells, being co-amplified with other proteins involved in the resistance to chemotherapy in cancer cells, as the ABC transporters ABCB4 and ABCB1. Sorcin overexpression results in increased resistance to many important anti-cancer drugs, and sorcin silencing is able to revert drug resistance. Sorcin is increasingly considered a useful marker of MDR and may represent a therapeutic target for reversing tumor multidrug resistance.

Sorcin regulates cardiac function, by modulating calcium channels and exchanger during cardiac contraction and, in particular, by participating in the termination of calcium-induced calcium release from the sarcoplasmic reticulum and in the relaxation processes.

Sorcin appears to be located at many important crossroads of human cells, and to have an important role on the onset of cancer, cardiac diseases and neurodegenerative diseases. Much has to be studied to continue to decipher the role of sorcin: e.g. the possible role in early development has to be studied, as that of sorcin in the nucleus, or the interaction and possible regulation of kinases. We feel that the next decade will bring new, exciting studies on this protein.
